# Implementation of Rapid Nucleic Acid Amplification Based on the Super Large Thermoelectric Cooler Rapid Temperature Rise and Fall Heating Module

**DOI:** 10.3390/bios14080379

**Published:** 2024-08-06

**Authors:** Jianxin Cheng, Enjia Zhang, Rui Sun, Kaihuan Zhang, Fangzhou Zhang, Jianlong Zhao, Shilun Feng, Bo Liu

**Affiliations:** 1 Xiangfu Laboratory, Jiashan 314100, China; chengjianxin001@163.com (J.C.); jlzhao@mail.sim.ac.cn (J.Z.); 2 State Key Laboratory of Transducer Technology, Shanghai Institute of Microsystem and Information Technology, Chinese Academy of Sciences, Shanghai 200050, China; z13753903091@163.com; 3 College of Chemistry and Materials Science, Shanghai Normal University, Shanghai 200234, China; 4 2020 X-Lab, Shanghai Institute of Microsystem and Information Technology, Chinese Academy of Sciences, Shanghai 200050, China; 5 School of Microelectronics, Shanghai University, Shanghai 200444, China; 6 Shanghai Si-Gene Biotech Co., Ltd., Shanghai 201899, China

**Keywords:** microfluidics, quantitative polymerase chain reaction (qPCR), proportional integral derivative (PID), thermoelectric cooler (TEC), Peltier effect

## Abstract

In the rapid development of molecular biology, nucleic acid amplification detection technology has received more and more attention. The traditional polymerase chain reaction (PCR) instrument has poor refrigeration performance during its transition from a high temperature to a low temperature in the temperature cycle, resulting in a longer PCR amplification cycle. Peltier element equipped with both heating and cooling functions was used, while the robust adaptive fuzzy proportional integral derivative (PID) algorithm was also utilized as the fundamental temperature control mechanism. The heating and cooling functions were switched through the state machine mode, and the PCR temperature control module was designed to achieve rapid temperature change. Cycle temperature test results showed that the fuzzy PID control algorithm was used to accurately control the temperature and achieve rapid temperature rise and fall (average rising speed = 11 °C/s, average falling speed = 8 °C/s) while preventing temperature overcharging, maintaining temperature stability, and achieving ultra-fast PCR amplification processes (45 temperature cycle time < 19 min). The quantitative results show that different amounts of fluorescence signals can be observed according to the different concentrations of added viral particles, and an analytical detection limit (LoD) as low as 10 copies per μL can be achieved with no false positive in the negative control. The results show that the TEC amplification of nucleic acid has a high detection rate, sensitivity, and stability. This study intended to solve the problem where the existing thermal cycle temperature control technology finds it difficult to meet various new development requirements, such as the rapid, efficient, and miniaturization of PCR.

## 1. Introduction

Clinical diagnostics and environmental monitoring both heavily depend on nucleic acid detection [1]. A novel instrument for nucleic acid extraction and detection has been made possible by the advancement of micromachining technology. This tool combines molecular diagnostics with microfluidic systems to expedite and simplify the procedure. The microfluidic chip technology exhibits notable attributes, such as miniaturization, low power consumption, high efficiency, and rapid analysis [2]. The issue of liquid transfer by conventional methods can be avoided with magnetically controlled microfluidic chip technology, which is a hotspot in the field of nucleic acid extraction and detection. This enhancement of the efficiency of nucleic acid extraction while preventing aerosol pollution is beneficial. More significantly, it can provide automatic control by combining washing, elution, detection, and nucleic acid extraction onto a single chip.

The advancement of medical health has led to the widespread application of quantitative polymerase chain reaction (qPCR) technology in various domains of life sciences, encompassing the medical diagnosis of infectious diseases and gene-based therapy [3]. The outbreak of COVID-19 has greatly increased the market demand for qPCR technology products. Quantitative PCR is the method by which a small sample of genetic material can be exponentially amplified and quantitatively measured in real time [4]. The PCR process necessitates the cycling of test samples through a temperature profile, typically at 95 °C, 55 °C, and 72 °C, multiple times. In order to obtain PCR effectively, the temperature of vessels with DNA samples must be changed quickly, repeatedly, and accurately in the thermal cycle system, which is usually performed in a PCR instrument. A typical 40-cycle PCR can take 2 h to complete. To overcome this, companies are developing novel thermal systems for qPCR testing [3]. The thermal cycle system is the fundamental and core component of the PCR instrument, which directly influences the gene amplification efficiency and reaction time. A traditional PCR instrument, the heating part of the thermal cycle system adopts a resistance wire, ceramic heater, microwave, etc.; the cooling part of the thermal cycle system uses a fan, compression refrigeration, etc.; and a lack of combined heating and cooling functions are evident [5]. Their physical heating and cooling rates are limited due to both the large thermal mass and the low thermal conductivity of the heat block. A thermoelectric cooler (TEC) is widely used in the thermal cycle system because of its characteristics of rapid cooling/heating switching, no noise, and high reliability. The TEC thermal cycling system based on the Peltier effect has gradually become a research hotspot.

qPCR technology has been developed to enable its use in point-of-care testing (POCT), where the test is administered, and results are obtained in a single visit to a health provider. The time required to complete a standard PCR protocol is becoming increasingly important for health providers; therefore, it is necessary to apply the temperature control algorithm and TEC to improve the temperature rise and fall rate and optimize the performance of the thermocyclers. TEC is a refrigeration method based on the thermoelectric effect (Peltier effect) of semiconductor materials; it is often used in advanced PCR thermal cycling systems. The Peltier effect occurs when the electrical current across the isothermal junction of two different semiconductor materials is connected in series to form a thermocouple, absorb heat, and release heat, which can be realized at both ends of the thermocouple, respectively. Changing the direction of the current leads to a cooling/heating effect at contact depending on the direction of the current flow and the sign of the Peltier coefficient [6]. The cooling/heating effect of TEC is closely related to its heat dissipation method (interlayer heat conduction, convection, and radiation) and structural design [7]. As the heat dissipation of TEC is equal to the sum of its cooling capacity and input power, the focus on solving the heat dissipation problem of TEC plays a crucial role in improving cooling efficiency [8]. A typical TEC module (figure) is composed of several pairs of P-type and N-type semiconductors (bismuth telluride). These two semiconductors are connected in series by a metal conductor (copper), and two insulators (ceramics) form hot and cold surfaces at both ends of the P-type and N-type semiconductors.

Due to the small thermal load of a single Peltier unit, the Peltier array adopts a large number of micro-miniature Peltier arrays. Therefore, the Peltier array structure has better heating/cooling efficiency, faster temperature rise and fall speeds, and system response time [9]. In this study, we intend to use the TEC temperature module prepared by the micro-Peltier array to heat and cool the PCR reaction system in the microfluidic chip. The fuzzy PID control algorithm is used to accurately control the temperature and achieve rapid temperature rise and fall (average rising speed = 11℃/s, average falling speed = 8℃/s) while preventing temperature overcharging, maintaining temperature stability, and achieving an ultra-fast PCR amplification process (45 temperature cycle time < 19 min). Since each model of TEC has different sizes, the layout of multiple TECs is also different; we adopted 2 × 2 (two rows and two columns, totaling four pieces) layout forms. In addition, in order to match the TEC ultrafast temperature cycle, a suitable DNA polymerase was also needed to achieve the ultrafast DNA synthesis process and ultimately achieve the rapid PCR amplification process.

## 2. Materials and Methods

### 2.1. Chip Design and Fabrication

**Figure 1:**
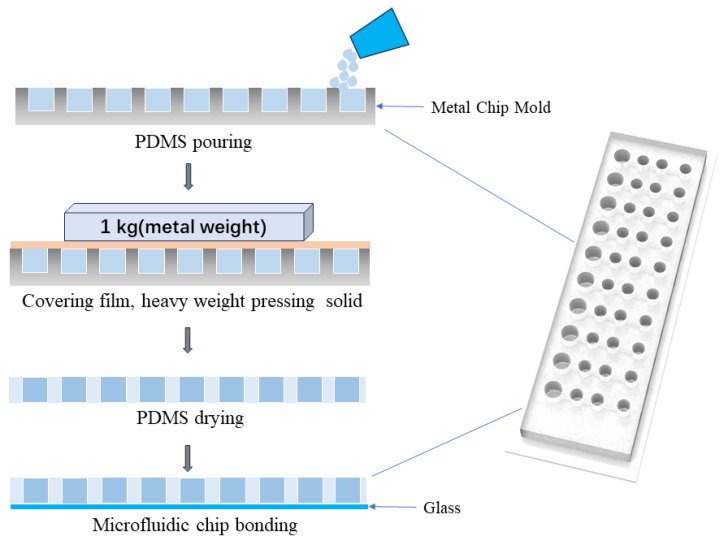
Microfluidic chip manufacturing process.

The biological microfluidic chip was made of metal mold and inverted mold. The polydimethylsiloxane (PDMS) is poured on a 10-sample hole metal mold and left to stand for ten minutes to remove surface bubbles. The surface of the poured PDMS is then covered with a new layer of PDMS film. Then, it is pressed with a metal weight, placed on a heating plate, and solidified at 85 °C for 3 h before removing the PDMS film after solidifying. The PDMS layer and the glass–silicon microcavity layer are then combined by oxygen plasma treatment. The integrated chip is then heated overnight in a 105 °C oven to recover the hydrophobicity of PDMS (Figure 1).

### 2.2. Reagent and Preparation

The extraction reagent (7.25 µL), protease K (0.25 µL), and magnetic beads (0.5 µL) were combined to create the lysis buffer (8 µL). Sangon Biotech (Shanghai, China) Co., Ltd. is where the magnetic beads were purchased. We acquired the protease K and extraction reagent from Shengxiang Biotech, Co. (Hunan, China). In order to prepare washing buffers 1 and 2, 50% ethanol and 13% PEG-8000 were utilized, respectively. In total, 10 μL of the RT-PCR amplification reagent was prepared by mixing 5.2 μL of the mix, 0.8 μL of the enzyme, and 4 μL of double-distilled water. The sequences of primers for the ORF1ab gene and TaqMan Probe are shown in Table 1. The synthetic plasmid PUC57, which was acquired from Sangon Biotech (Shanghai, China) Co., Ltd. and contains the SARS-CoV-2 ORF1ab gene, served as the template. Guangzhou Bondsheng Biotechnology Co., Ltd. (Guangzhou, China) purchased the synthetic SARS-CoV-2 pseudovirus. For the purpose of reagent separation and sealing, mineral oil (Fisher Scientific, Pittsburgh, PA, USA) was utilized as the oil phase. Amplification was performed according to the following procedure: 1 cycle at 50 °C for 3 min, 98 °C for 1 min, followed by 45 cycles at 98 °C for 1 s, and 58 °C for 5 s. The nucleic acid of the pseudovirus is RNA, which needs to be reverse-transcribed into DNA to be used as an amplification template; therefore, the reverse-transcription step was set up. Table 2 displays the PCR reaction process.

### 2.3. Nucleic Acid Extraction and Detection

Sequentially, 8 μL of lysis buffer, 4 μL of wash solution 1, 4 μL of wash solution 2, and 4 μL of the reaction solution were added to each chamber. The nucleic acids were adsorbed by the magnetic beads when 2 μL of the sample was added to the lysis buffer. The magnets drove the magnetic beads through the oil barrier into the washing chamber to wash away contaminants. Subsequently, the magnetic beads were pulled into the elution chamber for the reaction, and for five minutes, the nucleic acid was left to remain in the reaction buffer. Finally, the beads were pulled back into the front chamber to avoid interference with downstream detection. In addition, we carried out gradient extraction experiments at sample concentrations between 10^1^ and 10^3^ copies per μL to further assess the sensitivity and dynamic range. The exposure parameters for capturing the fluorescence images with an Olympus IX83 fluorescence inverted microscope were 5% power and 100 ms of exposure time. Detection was performed using the FAM fluorescence channel with an excitation wavelength of 485 nm and an emission wavelength of 524 nm. The fluorescence intensity of the exported photos was analyzed using ImageJ 3.82 software.

### 2.4. Thermo-Module

The multistage Peltier element has strong refrigeration performance, but it cannot achieve heating function; therefore, the single-stage Peltier was selected in this study, model number TEC1-19908AC (Qinhuangdao Fulianjing Electronics Co., Ltd., Qinhuangdao, Hebei, China). The following Table 1 describes the performance parameters of thermo-module resistance (Table 3).

### 2.5. Heat Pipe Radiator

**Figure 2:**
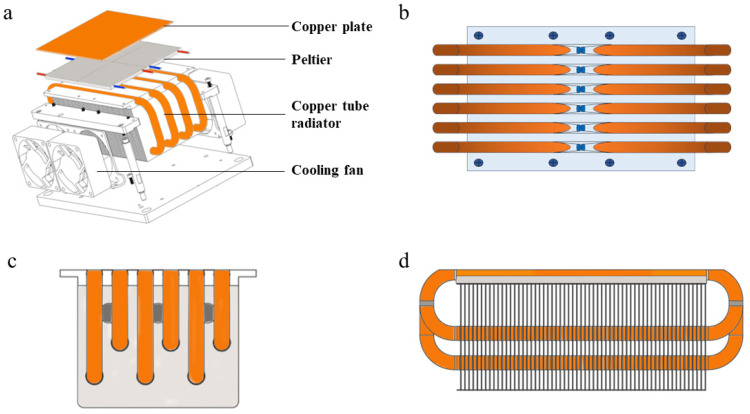
Fin-type heat pipe high-performance radiator. (**a**): Structure diagram of TEC; (**b**): heat pipe radiator top view; (**c**): heat pipe radiator side view; and (**d**): heat pipe radiator side view.

In this study, a fin-type heat pipe high-performance radiator was designed to improve the cooling efficiency of TEC and achieve the rapid rise and fall of the microfluidic PCR system (Figure 2a). The cavity-type heat pipe radiator substrate uses copper with higher thermal conductivity as the substrate material. Through grinding and polishing, the surface of the heat dissipation substrate has good flatness and smoothness, which fundamentally reduces the contact thermal resistance between the substrate and TEC. Multiple copper heat pipes are placed side by side and equidistant, and the copper pipes are embedded in the aluminum alloy fins. The strong cooling fan was imported from Delta (AFB0612EH) with a large air volume four-wire frequency converter. The size of the cooling fan was 120 × 120 × 38 mm (Figure 2b–d).

### 2.6. Temperature Control System

RS485 bus protocol: The RS485 bus adopts the half-duplex working mode, using balanced transmission and differential reception methods to achieve multi-point data communication. Due to the transmission line using twisted pair and differential input, the transmission line has strong resistance to common mode interference.

Main control circuit: the SMT32F103ZET6 chip is used as the core controller of the thermal cycle system, and the peripheral system circuit is designed to achieve functions such as PWM signal modulation and data communication and complete the overall control of the temperature control system.

Temperature control circuit: The power drive circuit is designed based on the principle of thermoelectric semiconductor refrigeration technology. The SMT32 control circuit outputs the PWM wave to adjust the power size. The main circuit adopts a Buck + H bridge circuit to achieve functions such as driving the semiconductor refrigeration chip, current commutation, and temperature control cycle.

Temperature detection circuit: The STM32F103ZET6 microcontroller uses the MAX31865 chip to read the PT1000 resistance value and collect data. The temperature measurement circuit converts the temperature value into a voltage signal and sends it to the microcontroller. The fuzzy PID control algorithm regulates the output signal to achieve the closed-loop feedback control of the system.

## 3. Results

### 3.1. The Biological Microfluidic Chip

**Figure 3:**
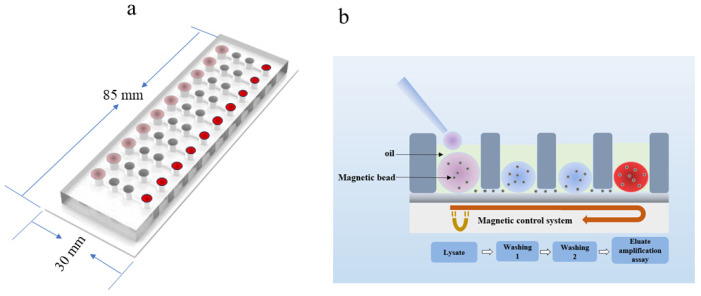
The biological microfluidic chip and schematic diagram of the nucleic acid extraction and purification processes. (**a**): The biological microfluidic chip front view. (**b**): Schematic diagram of the nucleic acid extraction and purification process.

The biological microfluidic chip has a longitudinal array of 10 reaction units. The chip mainly consists of a liquid storage chamber, microflow tube, sample addition hole, and glass base. The storage chamber includes a sample lysis chamber, washing solution chamber 1, washing solution chamber 2, and PCR amplification chamber, respectively. During the experiment, mineral oil is filled into the four chambers of the chip in advance, and then the lysis reagent, washing reagent, and amplification reagent are sequentially injected into the corresponding chamber in turn, and each reaction reagent is wrapped in an independent chamber by mineral oil (Figure 3a,b). In this study, we used SanMag Si-OH, which is made up of superparamagnetic Fe_3_O_4_ as the core, modified with the SiO_2_ layer on the outer surface and special non-specific adsorption treatment with a high specific surface area. The magnetic beads have good adsorption of nucleic acids under the ionic liquid salt system and can be quickly separated to release nucleic acids when the conditions are changed; it is an excellent tool for nucleic acid enrichment, extraction, and purification. Lysate and wash solutions contain higher concentrations of the ionic liquid salt system. The fourth chamber contains only the amplification reaction solution. Due to the hydrophilic nature of nucleic acids, the amplification reaction solution can elute nucleic acids from magnetic beads.

### 3.2. Temperature Control Module Structure

**Figure 4:**
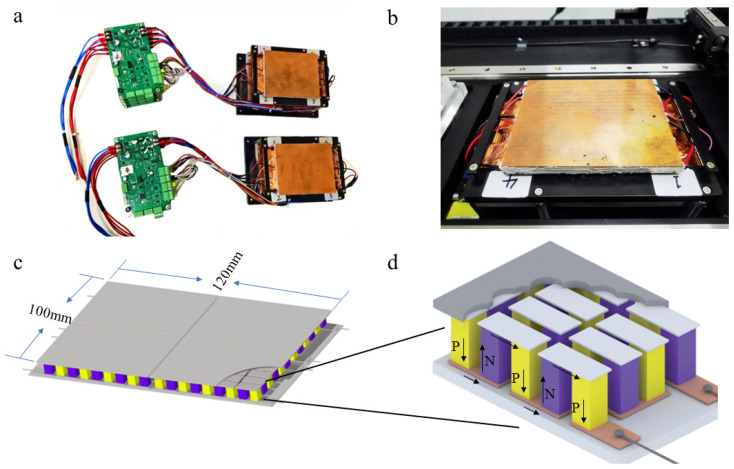
The structure of the thermoelectric cooler (TEC). (**a**,**b**): Physical drawing of TEC; (**c**): the temperature control console is composed of four TECs of the same size specifications; and (**d**): TEC combines semiconductor materials with thermoelectric effects to form P–N junctions.

In this study, a large TEC rapid rise and fall temperature control system based on the RS485 bus protocol was designed, including an interconnected temperature control console and control system. The temperature control console includes a semiconductor thermoelectric cooler and plate metal. A copper plate with a length width thickness of 120 mm × 100 mm × 2 mm is laid on the top of the semiconductor thermoelectric cooler, and the outlet edge of the metal plate is provided with a groove (Figure 4a). Platinum resistance temperature sensors are placed in each groove to collect the temperature of each semiconductor thermoelectric cooler. The lower surface of each semiconductor thermoelectric cooler is attached to the heat radiator, and a cooling fan is arranged on both sides of the heat radiator. In order to reduce the contact thermal resistance between the lower surface of TEC and the heat radiator, thermal conductive silicone grease was coated to the contact surfaces with uniform thickness and no bubbles. The temperature acquisition circuit is connected to a thermistor attached to the surface of the heat radiator, which is used to detect the real-time temperature of the heat sink in the heat radiator so that the heat sink can maintain a reasonable temperature range of 40–50 °C. The maximum temperature difference between the cold and hot surfaces of the TEC is 60 °C. If the maximum temperature required for the heating surface is 100 °C, the cold surface/radiator surface cannot be lower than 40 °C; otherwise, the heating surface may not reach the target of 100 °C. The cooling fan and heat radiator are fixed on the movement bracket assembly.

The working principle is that the heat generated by TEC is transferred to the heat dissipation substrate through thermally conductive silicone grease. After the heat dissipation substrate is heated, the heat is uniformly transferred to the heat pipe and then transferred to the outer wall of the heat pipe, and the fin bundles on the outer wall through thermal conduction. Finally, the heat is taken away from the heat dissipation fins through forced air cooling (Figure 4b).

### 3.3. Peltier Structure and Peltier Effect

The temperature control console is composed of four TECs of the same size specifications. The size of each TEC is 30 mm × 25 mm; the four TEC pieces can be combined to create a temperature control console (two rows and two columns, totaling four pieces) with a length and width of 120 mm × 100 mm (Figure 4c). Each TEC corresponds to a control board, and the four control boards are connected to each other through the RS485 bus protocol. Each control board consists of a microcontrol unit and H-bridge drive circuit connected to the microcontrol unit, a TEC temperature acquisition circuit, a heat sink temperature acquisition circuit, a fan control circuit, the USB to serial circuit and the RS485 bus circuit. The H-bridge driving circuit is used to drive the TEC for temperature rise and fall, which includes the TEC + interface and TEC- interface, connected with the positive and negative electrodes of the TEC, respectively. The TEC temperature acquisition circuit is connected to a platinum resistance temperature sensor placed in the groove to obtain the real-time temperature of each TEC surface.

In practical applications, TEC combines semiconductor materials with thermoelectric effects to form P–N junctions forming thermocouple pairs. Then, when a direct current is passed into the thermocouple pair, the thermoelectric element experiences the phenomenon of temperature decrease at one end and temperature increase at the other, which is called the Peltier effect. This phenomenon is used to convert electrical energy into thermal energy, which is the basic principle of the TEC (Figure 4d).

### 3.4. Control System

**Figure 5:**
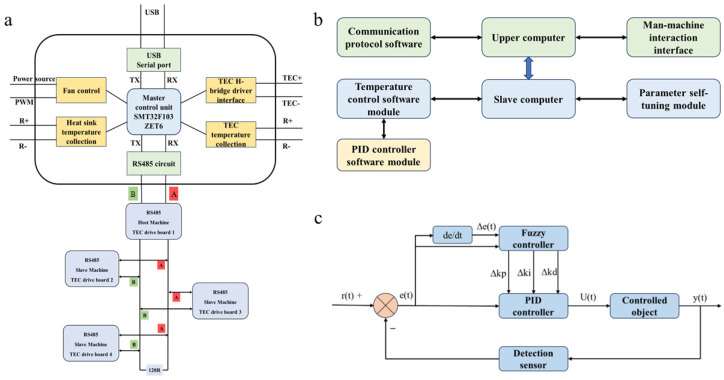
(**a**): The structure diagram of the temperature control board and one of the four control boards can be selected as the bus master, and the other three boards can be selected as slaves. (**b**): Temperature control system software overall design frame; (**c**): fuzzy PID control principle.

One of the four control boards can be selected as the bus master, and the other three boards can be selected as slaves. The bus master receives configuration parameters from the upper computer through the USB to the serial port circuit and transmits them to each slave computer through the RS485 bus. During the working process, each slave performs temperature control and sends real-time temperature to the bus master. In the temperature control process, the host computer synchronizes real-time temperature data with the slave to ensure that the temperature control of the four small TECs is synchronized with temperature rise and fall and the temperature error is maintained between 0.5 and 1°C. The H-bridge drive circuit is built using MOS transistors, which can be used to drive the TEC for the rising and falling temperature. The TEC temperature acquisition circuit uses the SMT32F103ZET6 chip and platinum resistance temperature sensor PT1000 as the thermistor digital output converter and adopts the bridge circuit as the temperature measurement circuit to complete the conversion of the resistance value to analog voltage. The temperature acquisition circuit of the heat sink and the IO of the ADC function in the microcontroller unit is used for partial pressure acquisition to detect the real-time temperature of the heat sink. The USB to serial port circuit is used to receive temperature configuration parameters from the upper computer, such as the maximum temperature of 95 °C, a low temperature of 60 °C, and 45 cycles of digital information (Figure 5a).

### 3.5. Temperature Control System

The overall design block diagram of the temperature control system is shown in Figure 5, which mainly includes the design of the human–computer interaction interface, the communications protocol of the main controller, and the data display, analysis, and storage module. The algorithm’s research mainly focuses on the fuzzy PID parameter setting module, PID controller software design, and temperature-accurate control module.

After setting the temperature parameters on the upper computer, the configuration parameters are transmitted to the host in the control board through the USB to the serial port circuit. After the host parses the parameters, the parameters are synchronously distributed to each slave computer through the RS485 bus. After the host and slave have analyzed the temperature parameters, which starts with temperature control, the TEC temperature acquisition circuit collects the surface temperature of the TEC in real-time. At the same time, the temperature acquisition circuit of the heat sink also collects the temperature of the heat sink in real-time. During the heating process, each slave transmits the current temperature in real-time to the host through the RS485 bus and maintains temperature synchronization with the host. At the same time, the host uploads the current temperature data in real-time to the upper computer through the USB to the serial port circuit, and the upper computer displays the temperature or draws the temperature curve (Figure 5b).

In order to achieve precise temperature control, the closed-loop control system was designed, and the fuzzy PID control met the demand for fast response. In process control, the combination of the PID controller and fuzzy controller can control the temperature accurately. The principle of fuzzy control is a way of manipulating the operation of the system through fuzzy reasoning and decision-making based on artificial knowledge and experience and transforming it into fuzzy language. In order to achieve the optimization and improvement of the PID control effect, in this design, the fuzzy control was combined with PID, and the PID controller completed self-adjustment through the defuzzification of the fuzzy controller. The input deviation of the fuzzy feedback controller is the preset rate of change r (t) and the incremental deviation e (t) obtained from the preset value of the fuzzy feedback output. The incremental deviation output by the fuzzy controller is ∆K_p_, ∆K_i_, ∆K_d_. In order to achieve the parameter self-tuning of K_p_, K_i_, and K_d_, the sum of the three parameters K_p0_, K_i0_, and K_d0_ of the PID controller was calculated (Figure 4c).

$$\left\{\begin{array}{l} K_{p}=K_{p0}+\Delta K_{p}\\ K_{i}=K_{i0}+\Delta K_{i}\\ K_{d}=K_{d0}+\Delta K_{d} \end{array}\right.$$

### 3.6. Temperature Cycle Test of TEC

**Figure 6:**
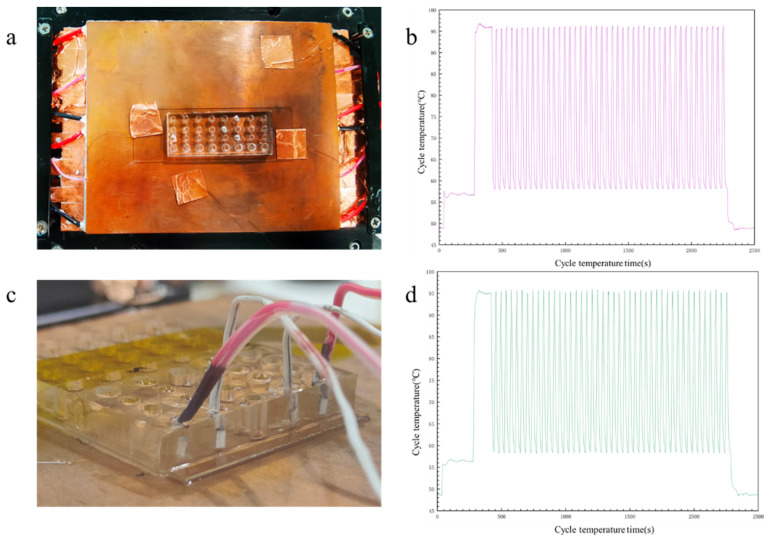
Temperature cycle test of TEC. (**a**,**b**): TEC surface temperature for 45 temperature cycles; (**c**,**d**): 45 temperature cycles of in-chip temperature.

The HH309A thermometer from OMEGA was used for the temperature test and temperature data acquisition. The measurement range of the HH309A thermometer was −200~1370 °C, the accuracy was ±0.4 °C at −200~200 °C, and the maximum error was ± 0.3 °C within 0~100 °C. The experiment simulated the PCR amplification process by affixing the thin copper plate as a load with thermal conductive adhesive on the Peltier and affixing the thermocouple to the upper surface of the copper block with 3M copper foil tape (to reduce heat exchange between the copper block and air) (Figure 6a,b).

We set the temperature for 58 °C at 60 s, 98 °C at 60 s, and 45 cycles of 98 °C at 3 s and 58 °C at 5 s in the software. The 45 temperature cycles took 700 s, with 58 °C rising to 96 °C for about 3.4 s when the heating rate was 11 °C/s and 96 ℃ falling to 58 °C for about 4.7 s with a cooling rate of 8 °C/s. Due to the exchange of space temperature, the highest surface temperature of TEC detected by the instrument reached 97°C, and the droplet temperature in the chip was 95°C, which reached the optimal temperature required for enzyme activity. As shown in the figure, the PCR amplification procedure of the TEC module was stable, and the temperature rise and fall was rapid, which quickly completed PCR amplification (Figure 6c,d).

### 3.7. Detection of Simulated Clinical SARS-CoV-2 Samples

**Figure 7:**
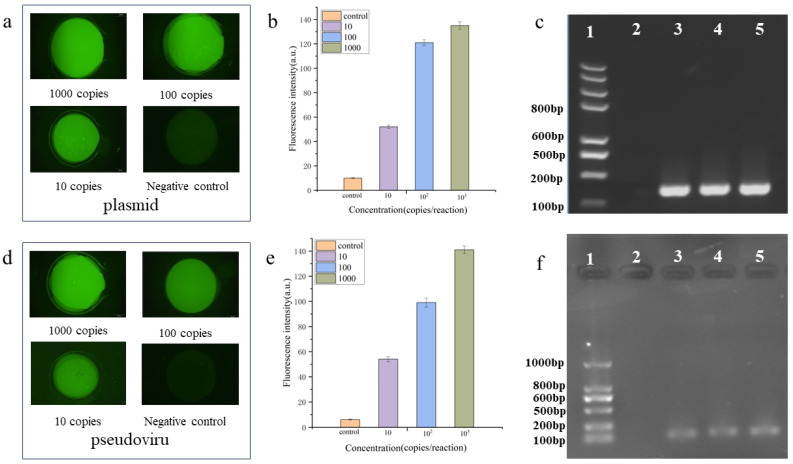
Testing the rapid amplification efficiency of the TEC and the detection performance of the integrated chip. (**a**,**b**): Plasmid containing the SARS-CoV-2 ORF1ab, and the concentration range from 10^3^ copies/µL to 10 copies/µL; (**d**,**e**): SARS-CoV-2 synthetic pseudovirus and the concentration range from 10^3^ copies/µL to 10 copies/µL; and (**c**,**f**): nucleic acid extraction integrity verification. Amplification products were analyzed with 2.5% agarose gel electrophoresis with lanes 3, 4, and 5 representing the gel electrophoresis images of nucleic acid amplification products obtained using on-chip and commercial kit methods, respectively, with a negative control (Lane 2) and marker (lane 1). The product length is 120 bp of the target length.

In order to test the rapid amplification efficiency of TEC and the detection performance of the integrated chip, we prepared a synthetic plasmid containing the SARS-CoV-2 ORF1ab and SARS-CoV-2 synthetic pseudovirus particles to simulate the actual samples. The concentration range of the series of synthetic virus particle samples ranged from 10^3^ copies/µL to 10 copies/µL. The entire process of nucleic acid extraction and purification to PCR amplification and digital detection was completed using the integrated chip, further validating the PCR amplification performance and sensitivity of TEC. The samples containing different concentrations of pseudoviral particles were added to the functional area of the integrated chip, and steps were followed according to the operation instructions for nucleic acid lysis, extraction, purification, and amplification. After purification, the samples were subjected to digital PCR amplification. The quantitative detection results of the SARS-CoV-2 pseudovirus and plasmid specific sequence (ORF1ab) from the system after the end of the TEC rapid thermostatic cycle (Figure 7a,b), and amplification products were analyzed with 2.5% agarose gel electrophoresis, and the result showed no false positives in negative control samples (Figure 7c). According to the different concentrations of added viral particles and plasmids, fluorescence signals of different intensities were clearly observed, and the assay was able to achieve a limit of detection (LoD) as low as 10 copies/µL (Figure 7d,e), and amplification products were analyzed with 2.5% agarose gel electrophoresis; the result showed no false positives in negative control samples (Figure 7f). These results show that the TEC with a fast temperature cycle and integrated chip can accurately detect simulated real samples of viral particles and has good specificity and sensitivity.

## 4. Discussion

Since the COVID-19 outbreak, pneumonia caused by the mixed infection of the COVID-19 virus, influenza A virus, and mycoplasma has emerged in succession. Due to the rapid spread of the virus, it is necessary to speed up the nucleic acid detection of personnel in high-risk areas. PCR amplification instruments have played a vital role in the process of virus detection [10]. PCR nucleic acid detection technology was one of the most commonly used detection methods during the outbreak of the epidemic and has demonstrated many advantages in the detection of infectious diseases. Although PCR is the gold standard for detecting pathogens in practical applications, it has drawbacks such as slow reaction speed and a long reporting cycle time. The lengthy rise and fall time in the reaction process of PCR is the main reason for this. With the development of high-tech and the advancement of medicine, in order to meet demand in people’s efficient and fast-paced lives, people are increasingly focusing on the miniaturization, simplified operation, and real-time reporting results of POCT devices. POCT is an on-site, real-time detection method that can achieve rapid and accurate detection results through the use of portable analytical instruments and matching reagents. The core of the POCT instrument is the thermal cycling system [11]. The TEC is widely used in thermal cycling systems due to its rapid cooling/heating switching, no noise, high reliability, and convenience [12,13]. The TEC using a thermoelectric cooler has been successfully applied in POCT for high flux dissipation under the Peltier effect. The application of TEC can shorten the PCR amplification time of the POCT instrument.

The TEC is composed of two different semiconductor materials (P-type and N-type) P–N junction; when there is a direct current passing through the P–N junction, due to the heat absorption or exothermic effect of the electrons and holes in the two materials during the process of movement across the P–N junction, the P–N junction shows a cooling or heating effect known as the Peltier effect [14,15]. The Peltier effect connects two different semiconductor materials into an electric couple and generates a temperature difference at the two junctions when a direct current is applied. Through experimental research, it has been found that an electric current can not only affects the direction of heat flow but also the size of the current, which is proportional to the intensity of Peltier heat. One important aspect of the Peltier effect is that the direction of the heat exchange at the junction (heating or cooling) can be controlled by switching the current’s direction. Peltier’s superior performance makes it capable of rapid temperature rise and fall. In this study, the Peltier heating module achieved 10℃/s heating speeds and 5℃/s cooling speeds. The characteristics of the rapid heating and cooling of Peltier can be applied to the POCT detection machine, and Peltier can not only greatly shorten the time required for the whole PCR amplification but also help to improve the efficiency of PCR amplification. In 2016, the temperature control experiment based on TEC was able to run on the Peltier effect, and on this basis, the characterization of the power semiconductor temperature control system was completed, which was initially applied to PCR instruments [16].

In this study, based on thermoelectric semiconductor refrigeration technology, we designed a thermal cycle temperature control system with a wide range, high precision, and fast rising and cooling rates. The temperature control unit adopts a microcontroller to control the hardware circuit, which can regulate the temperature change speed and temperature maintenance time of the semiconductor refrigeration chip. The temperature and time parameter values can be set through the PC end, and the temperature cycle range and number of cycles can be controlled, achieving the automatic temperature control process of gene amplification experiments. The core of a temperature control system is temperature control and control algorithms. In terms of temperature control, the use of thermoelectric semiconductor refrigeration technology, combined with high-power driving circuits, can achieve a good temperature hardware control system [17]. In terms of the temperature control algorithm, the fuzzy PID control algorithm can be used to improve the system performance indicators for the temperature overshoot, instability, and other phenomena that are prone to occur during the rapid temperature rise and fall cycle process [18]. In practical applications, the fuzzy PID control algorithm has the characteristics of a simple control principle, excellent reliability, convenient parameter adjustment, and no need for accurate models, making it the most frequently used industrial control algorithm [19,20]. The fuzzy PID controller is based on traditional PID and incorporates the fuzzy control theory, which stores the rules, operations, and PID parameters of the control system in the knowledge base. According to the controlled system, the appropriate control structure is selected, the fuzzy rules are settled in the field reasonably, effective and correct inference algorithms and decisions are carried out, and finally, automatic optimization and parameter adjustment are achieved [21,22].

The entire nucleic acid detection workflow, including sample processing, nucleic acid extraction and purification, and nucleic acid amplification and detection, is incorporated into the highly integrated and automated nucleic acid detection system. Applied primarily to pathogen infection identification, precision medicine, and infectious disease diagnosis, the integrated nucleic acid detection system is an essential tool for clinical diagnosis and therapy. One of the most crucial parts of the clinical diagnostic and treatment procedure is the qualitative or quantitative diagnosis of pathogens [23,24]. Various targets have been used for SARS-CoV-2 detection, such as the RNA-dependent RNA polymerase (RdRp) gene, nucleocapsid (N) gene, envelope (E) gene, spike (S) gene, and ORF1b or ORF8 regions of the SARS-CoV-2 genome [25,26]. In this study, we utilized the rapid temperature rise and fall performance of the TEC to achieve nucleic acid extraction, purification, and the amplification of the synthetic SARS-CoV-2 virus and plasmid containing the SARS-CoV-2 ORF1ab gene at different concentrations in microfluidic chips. Pseudoviruses are recombinant viruses whose genes are usually altered or modified virus-like particles with virus-mimicking physical structures and specific nucleic acid sequences, with analytical properties similar to those of real viruses but without the ability to self-replicate and infect; thus, they are capable of participating in the entire process of virus detection from extraction to amplification. They are biosafe and can be used as measurement standards for the validation and evaluation of viral nucleic acid qualitative and quantitative measurement methods, as well as laboratory quality control. Plasmids are constructed as recombinant DNA molecules by inserting the target gene into a vector DNA molecule. Pseudoviruses can mimic the process of nucleic acid extraction, washing, and purification, while plasmids are used directly in the amplification step. After the nucleic acid amplification reaction of the pseudovirus and the plasmid, fluorescence values showed that both fluorescence intensities were similar. This work used the SARS-CoV-2 pseudovirus to further validate the sensitivity and performance of the chip and TEC. The quantitative results are displayed in Figure X, where varying fluorescence signals can be seen based on the concentrations of additional viral particles added. It was possible to achieve an analytical detection limit (LoD) as low as 10 copies per μL in the negative control without any false positives. The outcomes demonstrate the great stability, sensitivity, and detection rate of nucleic acid amplification achieved by TEC.

## 5. Conclusions

This paper focuses on the temperature cycle of PCR, using the Peltier element that combines heating and cooling functions. The STM32 microcontroller is used as the core controller, and MAX31865 is used as the H-bridge driver. The PCR temperature control module is designed by combining the state machine and fuzzy PID algorithm. Through temperature measurement accuracy testing and temperature control testing, it has been demonstrated that the temperature control module designed in this paper has strong stability, a fast heating and cooling rate, and can shorten the PCR experiment cycle through the use of Peltier components.

## Figures and Tables

**Table 1:** Primer and MGB probe sequence for SARS-CoV-2 ORF1ab genes.

Gene	Primer	Sequence
	Gene sequence	CAATTTAGTAGCAGTTCCTACAGGCTACGTTGATACATCTAATGCAACAGAGTTTTCTAGGGTGAGTGCTAAACCACCACCTGGTGACCAATTTAAACATCTTATACCACTTATGTACAAAGGATTACCTTGGAACATTGTGCGTATAAAGATAGTTCAGATGTTAAGTGATACACTTAAAAACCTTTCAGACAGAGTCGTTTTTGTCCTTTGGGCACATGGTTTTGAGCTGACATCTATGAAATACTTTGTCAAAATAGGACCTGAACGCACTTGTTGCTTATGTGACAAACGTGCTACCTGTTTTTGCACAGCATCTGATACTTATGCGTGTTGGCATCACTCAGTTGGATTTGACTATGTCTACAACCCTTTCATGATTGATGTTCAACAATGGGGTTTTACTGGTAATCTTCAAAGTAACCATGACCAATACTG
ORF1ab gene	Forward primer	TAGCTAATGAGTGTGCTCAAGTATT
	Reverse primer	GTTGTGGCATCTCCTGATGAG
	TaqMan Probe	FAM-TGGTCATGTGTGGCGGTTCACTAT-MGB

MGB: Minor groove binder. An improvement in the Tm value of the probe and increase in the specificity of the hybridization reaction.

**Table 2:** PCR reaction program.

Procedure	Temperature	Time	Cycle Number
Reverse transcription	50 °C	3 min	1 cycle
Initial denaturation	98 °C	1 min	1 cycle
Denaturation	98 °C	1 s	45 cycles
Annealing/extend	58 °C	5 s	

**Table 3:** Performance parameters of Peltier.

Performance Parameters			Remarks
Resistance	2.28 Ω ± 10%		Note-1
Imax.	8.5A		Note-2
Vmax	24.1 V		Note-3
	Th = 27 °C	Th = 50 °C	Note-4
Qcmax	118 W	130 W	Note-5
ΔTmax	68 °C	75 °C	Note-6
Solder melting point	235 °C		Note-7
Compression strength	1 MPa		Note-8

Note-1, measured by AC 4-terminal method at 25 °C; Note-2, input current resulting in greatest ΔT (ΔTmax); Note-3, maximum DC input voltage at ΔTmax and Th = 27 °C; Note-4, the temperature of the TEC hot side during operation; Note-5, the maximum amount of heat that can be absorbed on the cold side (occurs at I = Imax, ΔT = 0 °C); and Note-6, the maximum temperature difference that TEC can achieve (occurs at I = Imax, Qc = 0 W). (ΔTmax are measured in a vacuum 1.3 Pa); Note-7, the lowest melting point of solder used in the thermoelectric module; and Note-8, recommended maximum compressive stress at the unit area.

## Data Availability

The original data and contributions presented in this study are included within the article. For further inquiries, please contact the corresponding author.
